# Effects of Vegetation Structure on the Location of Lion Kill Sites in African Thicket

**DOI:** 10.1371/journal.pone.0149098

**Published:** 2016-02-24

**Authors:** Andrew B. Davies, Craig J. Tambling, Graham I. H. Kerley, Gregory P. Asner

**Affiliations:** 1 Department of Global Ecology, Carnegie Institution for Science, 260 Panama Street, Stanford, California, 94305, United States of America; 2 Centre for African Conservation Ecology, Department of Zoology, Nelson Mandela Metropolitan University, Port Elizabeth, 6031, South Africa; Università degli Studi di Napoli Federico II, ITALY

## Abstract

Predator-prey relationships are integral to ecosystem stability and functioning. These relationships are, however, difficult to maintain in protected areas where large predators are increasingly being reintroduced and confined. Where predators make kills has a profound influence on their role in ecosystems, but the relative importance of environmental variables in determining kill sites, and how these might vary across ecosystems is poorly known. We investigated kill sites for lions in South Africa’s thicket biome, testing the importance of vegetation structure for kill site locations compared to other environmental variables. Kill sites were located over four years using GPS telemetry and compared to non-kill sites that had been occupied by lions, as well as to random sites within lion ranges. Measurements of 3D vegetation structure obtained from Light Detection and Ranging (LiDAR) were used to calculate the visible area (viewshed) around each site and, along with wind and moonlight data, used to compare kill sites between lion sexes, prey species and prey sexes. Viewshed area was the most important predictor of kill sites (sites in dense vegetation were twice as likely to be kill sites compared to open areas), followed by wind speed and, less so, moonlight. Kill sites for different prey species varied with vegetation structure, and male prey were killed when wind speeds were higher compared to female prey of the same species. Our results demonstrate that vegetation structure is an important component of predator-prey interactions, with varying effects across ecosystems. Such differences require consideration in terms of the ecological roles performed by predators, and in predator and prey conservation.

## Introduction

Predator-prey relationships play an integral role as top-down ecological controls, influencing ecosystem function and stability [1,2,3]. Imbalances in the ratio of predator to prey, or an absence of predators, can result in major regime changes and shifts in ecosystem functioning [4,5]. Yet, maintaining or restoring predator-prey relationships that are self-sustaining is challenging [6,7], especially within protected areas, which rarely encompass entire, self-sustaining ecosystems [8,9]. Globally, populations of large carnivores are experiencing sharp declines [10], and protected areas are rapidly becoming the last or only strongholds for many species [11]. Furthermore, in an attempt to restore ecosystem functioning and to increase tourism, managers of many small reserves reintroduce large predators into ecosystems from which they have been absent for decades, and into heavily fragmented and transformed landscapes [12,13]. Sustaining balanced predator-prey interactions in these reserves requires an understanding of the functional relationship between predators and their environment. The hunting habits of such predators are of particular interest because they largely determine the impact predators have on ecosystems, through effects on prey species distributions, abundance and behavior [4,14,15].

The 3D structure of vegetation is an important determinant of where predators make kills [16,17]. Vegetation structure, along with topography, is most relevant to predation through its influence on visibility, both in terms of predator concealment and prey detection of predators, as well as affecting the ability of prey to escape attack (see [18]). The area around an animal that is visible from the animal’s position (the animal’s “viewshed”) serves as a surrogate for both these processes because features that obstruct vision (such as vegetation or terrain) also hinder escape. Although viewsheds have previously been shown to influence predation sites [19], quantification of 3D vegetation structure and how animals perceive their surroundings is challenging in the field, and field-based measurements are difficult to translate into viewsheds (i.e. what an animal will actually be able to see). Many studies have relied on either rough measurements of expected predator viewsheds based on visual estimations by humans [20], or estimated viewsheds from vantage points such as rocky outcrops [19]. Alternatively, predators have been found to respond to broad proxies of vegetation cover, such as broadly classified vegetation types [21,22] or to visual estimates of shrub and grass cover made by humans [23]. Recent advances in Light Detection and Ranging (LiDAR) technology have enabled much improved measurements of vegetation structure applicable to animal ecology studies [24,25]. In particular, the 3D structure of vegetation, as viewed by predators and prey (their viewshed), has been found to be important in predator-prey relationships [16,17].

Vegetation structure can be modified by human and animal activities. Global change, through increased levels of CO_2_, is leading to bush encroachment, denser vegetation and wholescale biome shifts in many ecosystems (e.g. [26,27,28]), whereas deforestation results in more open habitats elsewhere [29]. Protected areas are not exempt from these drivers of landscape change, and conservation managers are similarly able to manipulate vegetation structure through modification of controls such as fire regimes [30] and herbivore densities [31]. Moreover, increasing elephant, *Loxodonta africana*, densities in South African protected areas are leading to large-scale tree loss and the opening up of vegetation [32,33]. An understanding of how such vegetation structural changes affect animal interactions, such as predator-prey relationships, is thus of increasing importance in global change, ecological and conservation contexts.

Lions, *Panthera leo*, are of particular interest in predator-prey studies because, when present, they are responsible for the majority of mammal herbivore mortality and biomass consumed for prey species larger than 10 kg [34]. They consequently perform important ecosystem roles through effects on populations, distributions and behavior of medium to large herbivores [14]. Lions usually hunt in groups [35] and are considered ambush predators, choosing prey ‘catchability’ over prey abundance and rely heavily on concealment during hunting [19]. Dense vegetation therefore provides cover for stalking lions and enables them to ambush their prey. However, although recognized as an important component of hunting success across a range of ecosystems [16,19,36,37], vegetation cover has been suggested as less important compared to other environmental variables for lions [23]. Other environmental variables important for ambush hunting, such as moonlight and wind orientation and speed also affect lion hunting success. Lions generally hunt more successfully on darker nights when it is easier to hide [23,36], and when wind orientation favors stalking [38], enabling them to approach downwind of and closer to potential prey. High wind speeds have been suggested to positively influence hunting success by making it harder for prey to hear predators over the increased noise of rustling vegetation [39]. However, the relative importance of these variables can differ across prey species, lion group sizes and ecosystems [23,36,37]. Wind speed, for example, was important in arid savanna environments, but only for select prey species [37], whereas it was inconsequential elsewhere [23]. Effects of different variables, and how vegetation structure interacts with them, are also likely to differ among prey species and prey sexes that employ different grouping and vigilance behavior. Female prey with young are generally more vigilant and tend to aggregate more compared to males and females without young [40]. Gregarious animals in general increase grouping patterns during risky conditions to increase their chances of detecting a predator [41]. How vegetation structure interacts with these variables, across varying ecosystems, to influence lion predation requires further examination.

Here, we investigated lion kill sites in relation to 3D vegetation structure, moonlight and wind in the subtropical thicket ecosystem of the Addo Elephant National Park, South Africa. The vegetation here varies from dense, impenetrable stands of thicket that preclude movement of even large bodied species [42], to open grasslands cleared for previous agricultural use, thus providing an opportunity to investigate predation patterns across a broad range of vegetation densities. Lion kill sites were located over four years using GPS telemetry data, and compared to sites where lions had been present over an extended period of time, but not made a kill, as well as to randomly distributed sites within the lion ranges that represented the full spectrum of vegetation densities available. We tested whether i) vegetation structure influences lion kill sites, and, for comparison with previous analyses of lion kill sites using LiDAR [16], if patterns differed between lion sexes in this markedly different ecosystem, ii) if vegetation structure is the primary environmental driver of lion kill site locations compared to other environmental variables, and iii) whether characteristics of kill sites were consistent across prey species and prey sexes.

## Materials and Methods

### Study site

**Fig 1:**
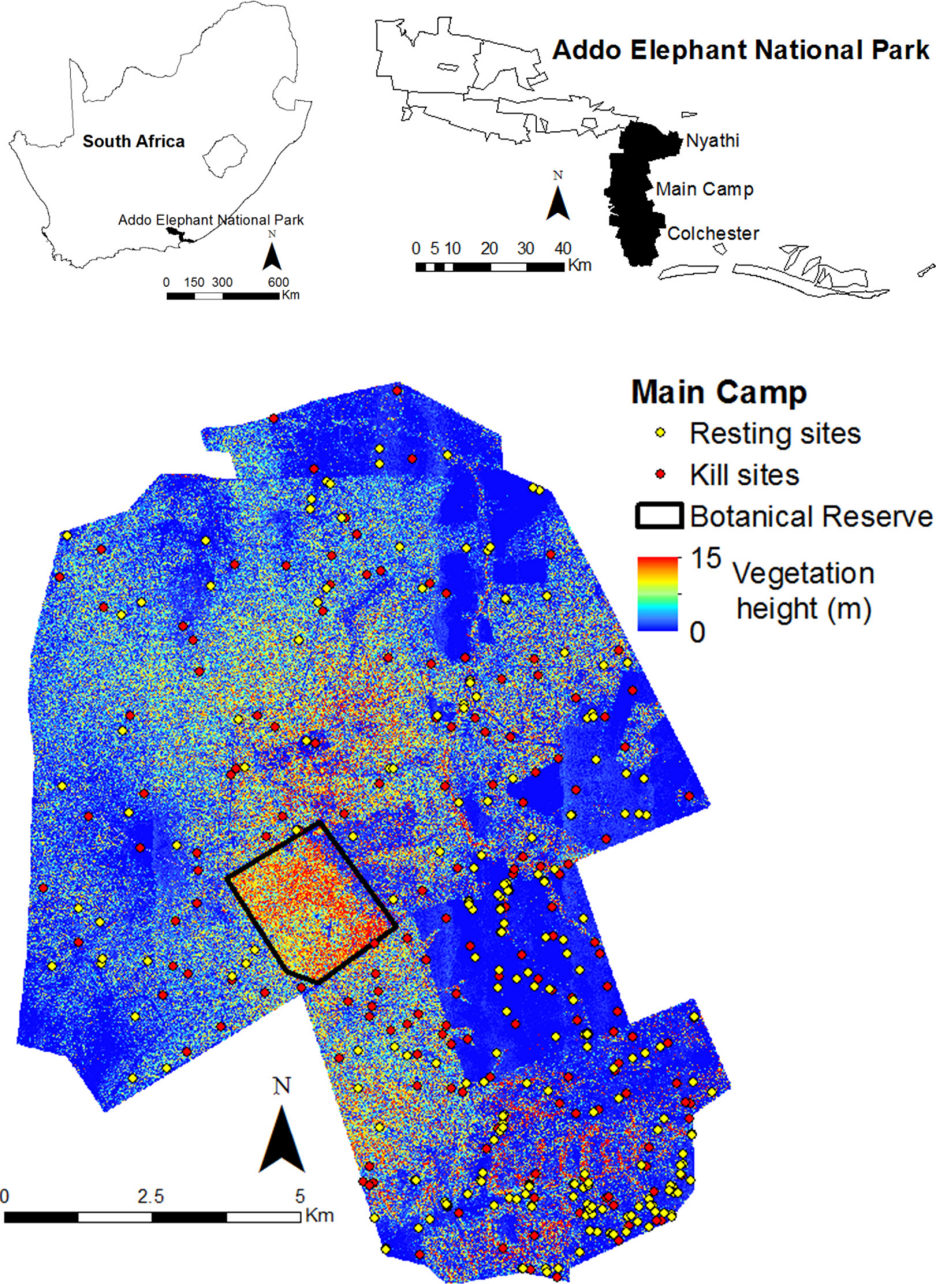
Map of the study site showing the location of the Addo Elephant National Park within South Africa (upper left panel), the Nyathi, Main Camp and Colchester sections within the park (upper right panel) and lion resting (non-kill) and kill sites within the Main Camp section (lower panel). Vegetation height derived from the LiDAR is also shown for Main Camp, as well as the botanical reserve, which lions do not have access to.

The study was conducted in the Addo Elephant National Park, South Africa (Addo, 33°31' S, 25°45' E), and focused on three adjacent sections: Main Camp, Colchester and Nyathi (Fig 1). Main Camp and Colchester form one contiguous area of ~28000 ha, whereas Nyathi is immediately north of Main Camp, but fenced separately and covers ~14000 ha. All three sections are dominated by succulent thicket vegetation, which is typically evergreen and dense, reaching between two and four m tall [42]. Thicket density varies across the park as a result of an opening up of the vegetation by differing densities of elephants over the last 50–60 years [32]. Interspersed between the thicket vegetation are large open grassy areas, which are remnants of previous agricultural activity. Main Camp and Colchester comprise a series of low, undulating hills, whereas Nyathi consists of flat plains in the south and parts of the rugged Zuurberg Mountains in the north. The region is semi-arid with annual precipitation ranging between 260 and 530 mm. Various artificially pumped water points provide water throughout the year [43]. The recorded prey community ranges from small antelope (e.g. Cape grysbok, *Raphicerus melanotis*, and common duiker, *Sylvicapra grimmia*) to large herbivores (e.g. buffalo, *Syncerus caffer*, and eland, *Tragelaphus oryx*) and comprises at least 10 species [44]. South African National Parks provided the permits necessary to conduct the fieldwork and airborne surveys for the study.

### Lion telemetry and field data

Lions were reintroduced into the Main Camp section of Addo in 2003 after an absence of over 100 years [12], and accessed the Colchester section in 2010 when the fence between the two sections was removed [45]. Lions were reintroduced into Nyathi in 2011. GPS telemetry and kill data from six lions (three females and one male in Main Camp-Colchester and a female and male in Nyathi), were collected between July 2010 and July 2014. Although the number of individual lions (particularly males) was relatively low, it constitutes all the lion social groups in Nyathi at the time of the study and most (~70%) of the lion social groups in Main Camp-Colchester. We were thus able to investigate lion foraging behavior for the majority of the Addo ecosystem lion population. Lions have been reintroduced to at least nine protected areas within the broader thicket biome ([12], lions have also since been reintroduced to Ibamba and Amakhala Game Reserves), with the Addo population representing one of the oldest populations in the region. Lions were captured and fitted with GPS collars (GPS/GSM and GPS/Satellite units, African Wildlife Tracking, Pretoria, South Africa) by veterinarians employed by South African National Parks using standard lion capture techniques [46] and chemical immobilization following their comprehensive ethical guidelines and in accordance with the Standard Operating Procedures of South African National Parks. South African National Parks approved all collaring procedures used in this study. Collars were set to download between three and five GPS locations per day (periodically some collars were programmed to record more locations per day). Using the movement data from the collars, clusters of GPS positions where lions were stationary within a defined proximity (<100 m) for more than two consecutive GPS locations were identified as potential resting or kill sites and investigated on foot to determine whether the lions made a kill or not at each GPS cluster. Predictions from GLM models were used to select clusters that were more likely to be kill sites, with priority placed on investigating these, and additional low probability clusters found in the immediate vicinity [47].

Kills were identified and classified (to prey species, prey sex and prey age class) through forensic investigation of GPS cluster sites (i.e. the presence of carcass remains such as bones, hair, horns or teeth) and the exact position of the kill was identified by the presence of stomach contents when available (n = 274). When stomach contents were not found, the site of the carcass remains was used as the kill site (n = 131). The stomach contents location is often considered diagnostic of the actual kill site because lions regularly move the carcass from the kill site [48], resulting in frequent separation of the carcass remains and stomach contents, whereas the stomach contents are almost always deposited within a few meters of the actual kill (P. Funston, pers. comm.). However, results of an analysis using only kill sites where stomach contents were present were comparable to one using kill sites when either stomach contents or only carcass remains were found (S1 Appendix). We thus included kill sites with only carcass remains present as well as kill sites where stomach contents and carcass remains were found in our final analysis. Scavenging in Addo is relatively rare, even for spotted hyenas, *Crocuta crocuta*, [49] and so carcasses found were assumed to have been killed by lions and not scavenged (from either hyenas or other lions). Furthermore, the dense vegetation and absence of vultures in Addo, likely makes it harder for predators to locate carcasses, both by sight and smell. Cheetahs, *Acinonyx jubatus*, are absent and leopards, *Panthera pardus*, are very rare in Addo (with 10 sightings recorded from ~11 000 camera trap days over six years (C. J. Tambling, *unpublished data*)) and not considered resident predators. Scavenging from these predators is therefore highly unlikely. However, because our study did not rely on direct observations of lions killing, we cannot definitively rule out male lions scavenging from female lions or other predators. We do however assume that the majority of female lion kills are a result of their own hunting [38,48,50]. Moreover, sex based differences were assessed primarily as a comparison to other LiDAR based lion kill site studies [16].

If no carcass remains were found, lions were assumed to be resting, possibly in unfavorable conditions to initiate a hunt or recovering from a failed hunting attempt. To reduce the risk of failing to detect carcass remains and, thereby, misidentifying kill locations as resting locations (false negatives), selection of resting clusters was restricted to those investigated within 100 days of their occurrence. After this period, success in detecting kills decreases (S1 Fig). Because false positives are far less likely to occur (due to the required presence of carcass remains), clusters that were checked after 100 days and found to be kill sites (i.e. with carcass remains) were included in the analysis.

The GPS cluster sites (kill and resting sites) used for analysis were further restricted to those where the most recent lion location data point was recorded less than 11 hours prior to the start of the cluster that reflects the presence of that lion. This ensured that weather conditions were likely to reflect the period prior to a kill or resting period. Eleven hours was chosen as a maximum because this was the longest scheduled interval between downloads, thus any interval longer than 11 hours was a result of the previous location point not being obtained. Time intervals between clusters and the previous point ranged from 2 to 11 hours, with a mean of 7 hours (SE = 0.09). We controlled for lions resting in the shade during the day (where viewsheds are smaller and distance to cover shorter (see [16]) by restricting the dataset (GPS cluster sites) to clusters that began between the hours of 17:00 and 08:00 (S2 Fig). Our final dataset consisted of 882 investigated GPS cluster sites, of which 405 were kills and 477 were resting sites or failed hunting attempts (Table 1).

**Table 1:** Sample sizes and proportion of each prey species killed. The number of kill and non-kill site locations used in the overall analysis.

Lions	Kill sites	Non-kill sites
All	405	477
Male	112	246
Female	293	231

### LiDAR and weather data

We mapped the full extent of the three Addo sections (Main Camp, Colchester and Nyathi) with discrete-return airborne LiDAR in March 2014 using the Carnegie Airborne Observatory (CAO [51]). The CAO LiDAR subsystem provides three-dimensional structural information of vegetation canopies and the underlying terrain surface. The GPS-IMU subsystem provides three-dimensional position and orientation data for the CAO sensors, allowing for highly precise and accurate projection of LiDAR observations on the ground. For this study, the CAO data were collected from 2000 m above ground level, using a scan angle of 36° and a side overlap of 50%, providing maps of ground elevation, woody canopy height and three-dimensional structure at 1.0 m spatial resolution (cell size of the derived measurements). LiDAR measurements of vegetation height were field-validated in early July 2014, and linear regression indicated a strong positive relationship between vegetation height measured in the field and with the LiDAR (*r*^2^ = 0.90, *P* < .001). Horizontal and vertical error estimates were 16 and 7 cm RMSE, respectively. Although the vegetation structure may have changed over the course of the three years preceding the LiDAR data collection, we assumed that because perennial woody biomass accounts for the majority of the thicket vegetation, the general structure of the vegetation would be largely unchanged over this time scale. Furthermore, because the thicket vegetation is evergreen and rainfall occurs throughout the year in Addo [32], seasonal differences in vegetation structure will be negligible.

**Fig 2:**
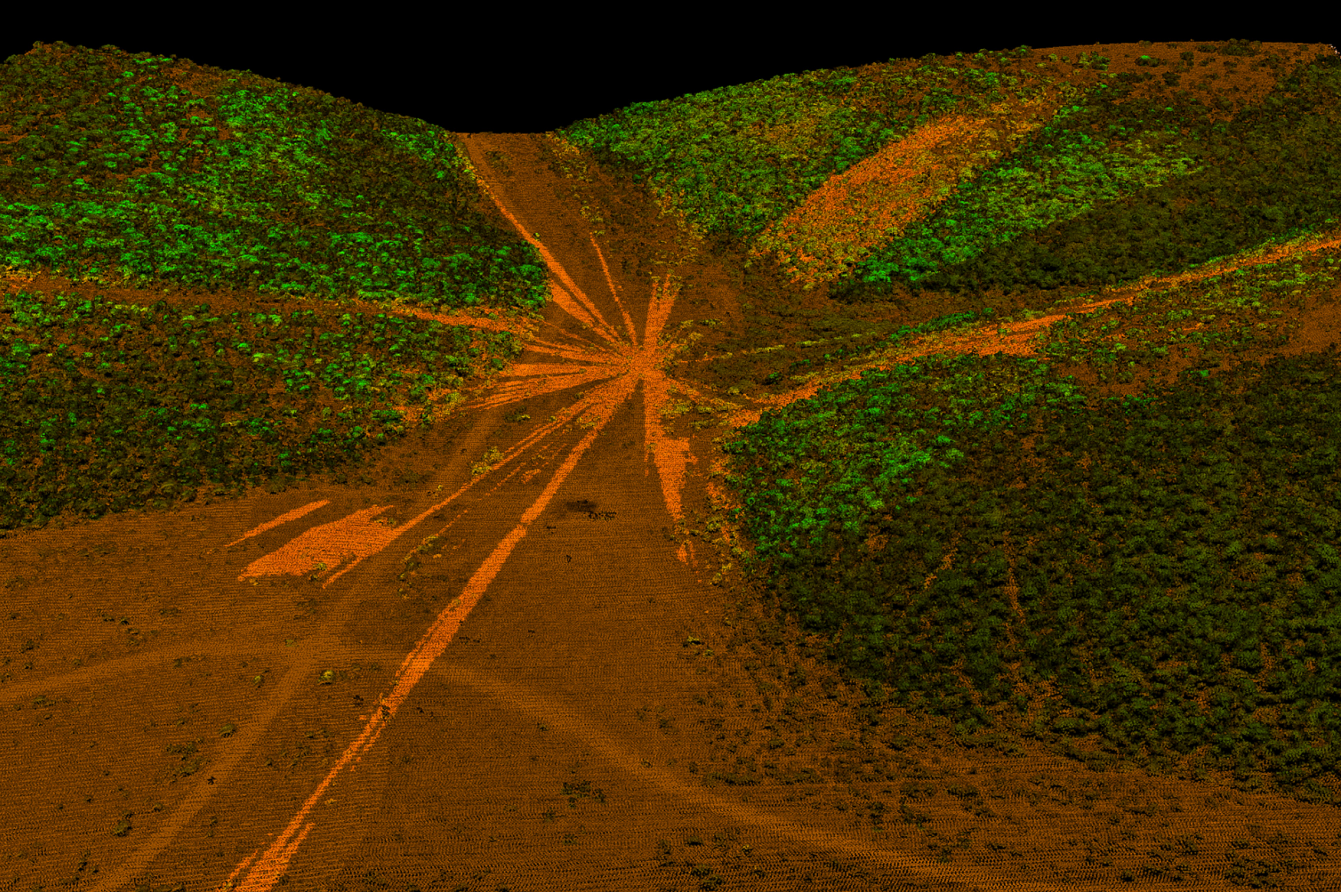
Example of a viewshed modelled from airborne LiDAR. The lighter colors indicate areas that are visible and accessible in all directions from the location of a central GPS cluster. Lighter orange represents visible terrain whereas lighter green represents visible vegetation. Shaded (darker) colors represent areas that are occluded from view. Parts of the landscape that are farther can be visible even though closer areas are occluded from view by objects in the foreground.

From the LiDAR data, we constructed ground and canopy digital elevation models (DEMs) and from these modelled the viewshed by calculating the area that was both visible and accessible (area that predator or prey were capable of occupying) from each kill and resting site. In addition, we calculated viewsheds around 329 randomly generated sites to compare characteristics of kill and resting sites to those randomly distributed in the 95% kernel home range of the lions. Initially, 441 (a sample size mid-way between the number of kill and resting sites) random sites were generated for the entire study area, with those falling outside of the lion use areas, excluded from the random dataset. For all viewsheds, we used a predator view of 0.5 m above the ground, approximating the height of a stalking lion’s head, and a prey view of 1.0 m, approximating the eye level of most available prey species. Any visible pixels within tree canopies above 0.5 m were excluded since they cannot be occupied by potential prey. Below canopy area that was visible at this height was included in the calculations. Both vegetation and terrain may obstruct sightlines (Fig 2). The area visible (i.e. the viewshed) was then calculated within a 50 m, 50–100 m, 100–300 m and 0–300 m radius around each point (except for random sites where the analysis was restricted to a 50 m radius–see analysis below). We limited the maximum extent of the viewshed to 300 m because this is the maximum distance from which lions initiate stalking hunts [52]. We divided the viewshed radius into several categories to investigate whether differences in viewsheds of increasing distance were relevant to where lions made kills. Because of the often hard boundaries between dense thicket vegetation and open plains in Addo, we further calculated the minimum distance to cover (potential ambush site) from each lion GPS cluster to account for sites that had large viewsheds, but were close to cover. We similarly calculated the minimum distance to cover around each cluster in four quadrants: 316–45° (north), 46–135° (east), 136–225° (south), 226–315° (west), and matched these to the prevailing wind direction during the time between the most recent GPS point before the cluster began and the start of the cluster in order to measure the minimum distance to cover downwind from each cluster.

Hourly weather data were obtained from the South African Weather Service (Addo weather station, approximately in the center of the study site) for wind speed and direction. Moonlight illumination, moonrise and moonset were extracted from a sunrise/moonrise website (http://www.timeanddate.com/moon/, Accessed: 18 August 2014) and sunrise and sunset times were estimated using an algorithm based on the latitude of the study site. Because we did not know the exact time a kill took place, wind speed data were averaged over the hours preceding each GPS cluster (from the GPS point prior to the beginning of the cluster to the start of the cluster). Similarly, we considered moonlight as a binary variable because we did not know the exact time of each kill, with moon either up or down at the start of the cluster, regardless of the moon phase. Moonlight was assigned as present for clusters beginning before (early evening) or after (early morning) dark. In addition, we investigated the influence of moonlight based on the average moon illumination (referenced by the proportion of the moon visible at each hour) before the cluster (since the previous GPS point), illumination at the start of the cluster and the minimum illumination (darkest time) since the end of the previous cluster (thus taking into account all possible times that the cluster could have started). We compared these conditions for kill and resting sites and found similar differences between these states for all metrics of moon illumination (S3 Fig). We chose to use moonlight as a binary variable (present or absent) at the start of the cluster because of uncertainties regarding kills that may have occurred during cloudy conditions when moonlight would be obscured, or in the early evening or morning when some sunlight might have been present. Simple presence or absence of moonlight is considered less fastidious, and patterns between different metrics showed similar differences between kill and resting sites.

### Analysis

All statistical procedures were conducted in R software, version 3.1.2 [53]. Collinearity between independent variables was assessed prior to analysis using variance inflation factors (VIF) and Spearman rank correlation tests [54]. High levels of correlation were found between the viewshed measurements in the different distance bands (0–50 m, 50–100 m, 100–300 m and 0–300 m), as well as between the viewsheds and the minimum distance to cover (both the distance in relation to wind direction and the absolute minimum distance) (S4 Fig). Therefore, we only used the viewshed in the 0–50 m distance band in the analysis. However, we retained the use of minimum distance to cover (in relation to wind direction) in some of the figures because it provides a slightly different, but important perspective in the context of the study site. Moreover, it provides a more direct comparison to previous work [16]. All variables had VIF values below two in the final statistical models. Viewshed was rescaled, multiplied by 10^−3^, to ensure similar scales between predictor variables in the models.

The analysis was performed in two stages. First, we investigated where lions made kills for all prey species, and whether kill site characteristics varied between lion sexes. We constructed a candidate set of 13 generalized linear models with binomial error distributions and logit link functions to examine relationships between state (kill or non-kill/resting) and lion sex, viewshed in the 0–50 m distance band, average wind speed before the start of the cluster and whether the moon was present or absent at the start of the cluster, as well as relevant two-way interactions following *a priori* hypotheses (Table 2). Because we had a larger sample size of female lion kills relative to resting sites compared to male lions, we subsampled our data without replacement to create equal sample sizes of kills to resting sites for male and female lions. This was achieved by taking a random subsample of the dataset containing 75 samples of each group: male kill sites, female kill sites, male resting sites, and female resting sites. This procedure was iterated 10 000 times with the candidate set of models applied to each subsample of data. Model selection was then performed by ranking each model according to the mean of the Akaike weights and the frequency each model obtained the lowest sample-size-corrected AIC*c* score over the 10 000 iterations [55]. Model averaging was then performed using the coefficients from the models that constituted a cumulative Akaike weight of 0.95 (Table 2) following the same subsampling procedure. The averaged coefficients were then used to examine effects of the different predictors and to make predictions from the regressions of which variables were most influential in characterizing kill and non-kill sites. Predicted effects (of whether a site was a kill or not) were visualized by varying the predictor variable in question across its range of values while keeping all other predictor variables constant at their mean. Differences in viewshed area between kill, resting and random sites within lion ranges were also assessed using a Kruskal-Wallis test with a *post-hoc* Pairwise Wilcoxon Rank Sum test (with a holm correction) to assess pairwise comparisons.

**Table 2:** The set of 13 candidate regression models applied to lion kill sites and ranked according to the frequency (Min AIC*c* frequency) each model received the minimum second order Akaike Information Criterion (AIC*c*) score and the Akaike weights (*w*_i_) of the models. The models (cumulative Akaike weight < 0.95) used in the model averaging techniques appear in normal font and the top ranked model is in bold. Models in italics were not used in model averaging.

Rank	Form of regression model	Mean AIC_*c*_	Min AIC*c* frequency	*w*_i_
**1**	**Viewshed + Wind speed**	**401.97**	**3986**	**0.275**
2	Viewshed	402.79	3516	0.226
3	Viewshed + Wind speed + Moonlight	402.65	1326	0.189
4	Viewshed + Moonlight	403.56	993	0.150
5	Viewshed + Wind speed + Moonlight + Sex + Sex: Viewshed	406.06	49	0.039
6	Viewshed + Wind speed + Moonlight + Sex + Sex: Moonlight	406.04	47	0.039
*7*	*Viewshed + Wind speed + Moonlight + Sex + Sex*: *Wind speed*	*406*.*06*	*38*	*0*.*039*
*8*	*Viewshed + Wind speed + Moonlight + Sex + Viewshed*: *Wind speed + Viewshed*: *Moonlight*	*407*.*53*	*37*	*0*.*021*
*9*	*Viewshed + Wind speed + Moonlight + Sex + Sex*: *Viewshed + Sex*: *Wind speed + Sex*: *Moonlight*	*408*.*95*	*7*	*0*.*012*
*10*	*Viewshed + Wind speed + Moonlight + Sex + Sex*: *Viewshed + Sex*: *Wind speed + Sex*: *Moonlight + Viewshed*: *Wind speed*	*411*.*93*	*1*	*0*.*004*
*11*	*Wind speed*	*417*.*75*	*0*	*0*.*003*
*12*	*Wind speed + Moonlight*	*418*.*51*	*0*	*0*.*002*
*13*	*Moonlight*	*418*.*73*	*0*	*0*.*002*

Second, we investigated whether there were any kill site characteristics specific to the different prey species and whether all or only some species kill sites differed from the non-kill/resting sites. Potential kill site differences for prey species sexes were also examined, with juvenile animals combined with adult females since they associate together. We only included prey species with at least 14 known kills (buffalo, eland, red hartebeest, *Alcelaphus buselaphus*, kudu, *Tragelaphus strepsiceros*, ostrich, *Struthio camelus*, warthog, *Phacochoerus africanus*, and zebra, *Equus quagga*, Table 3). For these analyses, generalized linear models with quasipoisson error distributions (because data were overdispersed) and logit link functions were constructed for each predictor variable that was influential in characterizing kill sites, i.e. viewshed within the 0–50 m distance band, the minimum distance to downwind cover and the average wind speed before the kill was made. These were now treated as response variables and relationships between them and prey species, prey sex and the interaction between these two variables examined. Models with and without the interaction term were compared using analysis of variance tables and likelihood-ratio tests. Where model fit was not significantly improved with the interaction present, it was removed from the model (the interaction term was retained only for minimum distance to cover). The non-kill sites were set as the reference level from which contrasts were made for each prey species (to detect which species kill sites differed to the non-kill sites). To detect kill site differences between prey species, the models were re-run without the non-kill sites present and multiple comparisons of means *post hoc* testing with Tukey contrasts used to examine pairwise comparisons. Non-kill sites could not be retained in the same model because of the lack of prey sex in the non-kill data. Differences between kill site characteristics for prey sexes were tested for each prey species individually using Wilcoxon Rank Sum tests for each of the three response variables.

**Table 3:** The number of kill site locations used in analysis of specific prey species and prey sexes.

Prey species	Males	Females and juveniles	Total
Buffalo	8	7	15
Eland	20	21	41
Hartebeest	35	20	55
Kudu	47	28	75
Ostrich	57	16	73
Warthog	8	6	14
Zebra	7	11	18

## Results

### Lion kill sites

**Fig 3:**
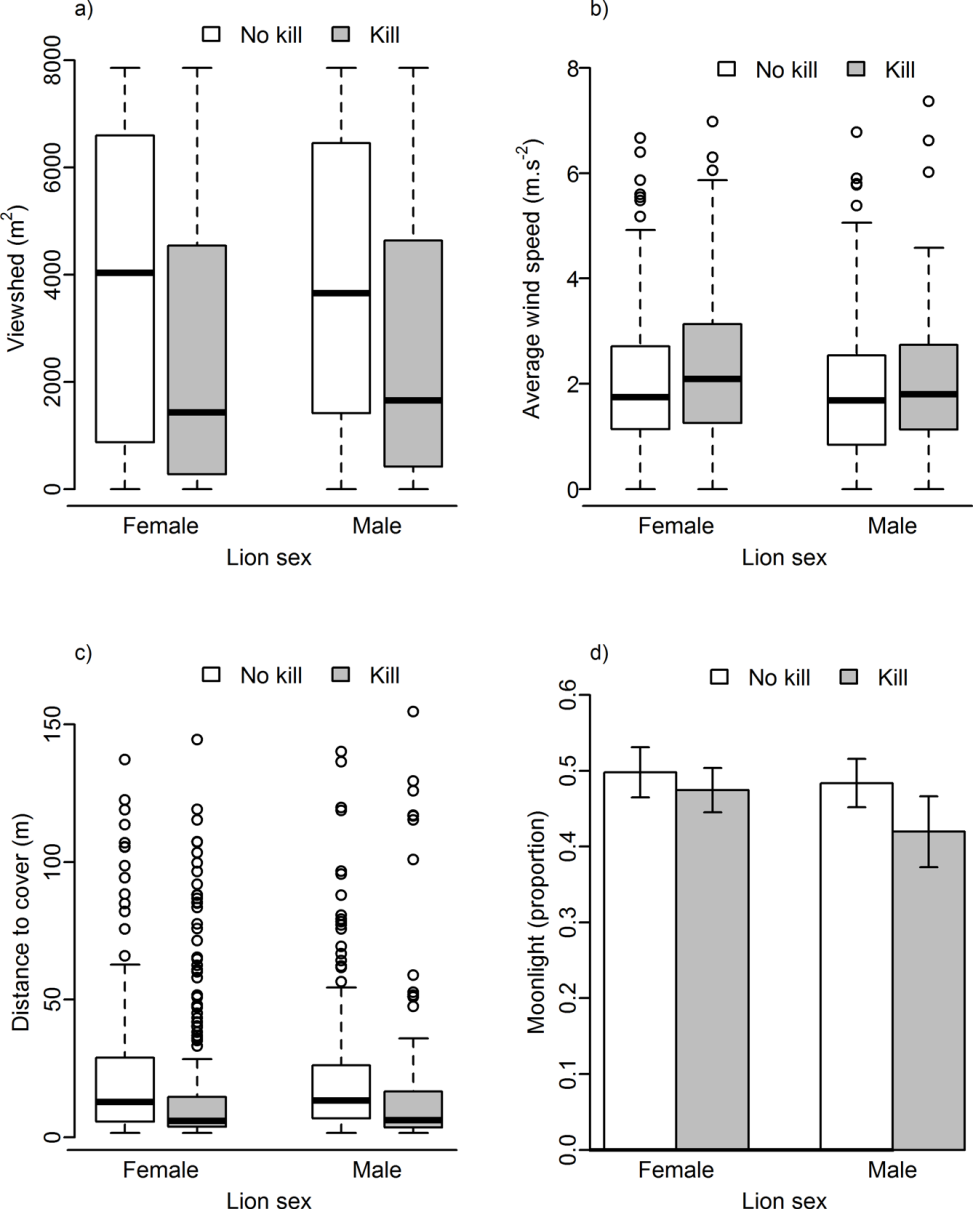
Lion kill and non-kill sites in relation to a) the size of the viewshed, b) the average wind speed before the start of the GPS cluster, c) the distance to the nearest cover (ambush site) relative to the prevailing wind direction and d) moonlight at the start of the cluster of the moon (1 = moon present, 0 = moon absent). No interaction terms between vegetation and weather variables were significant in predicting the locations of kills. Error bars in d) represent standard error.

**Fig 4:**
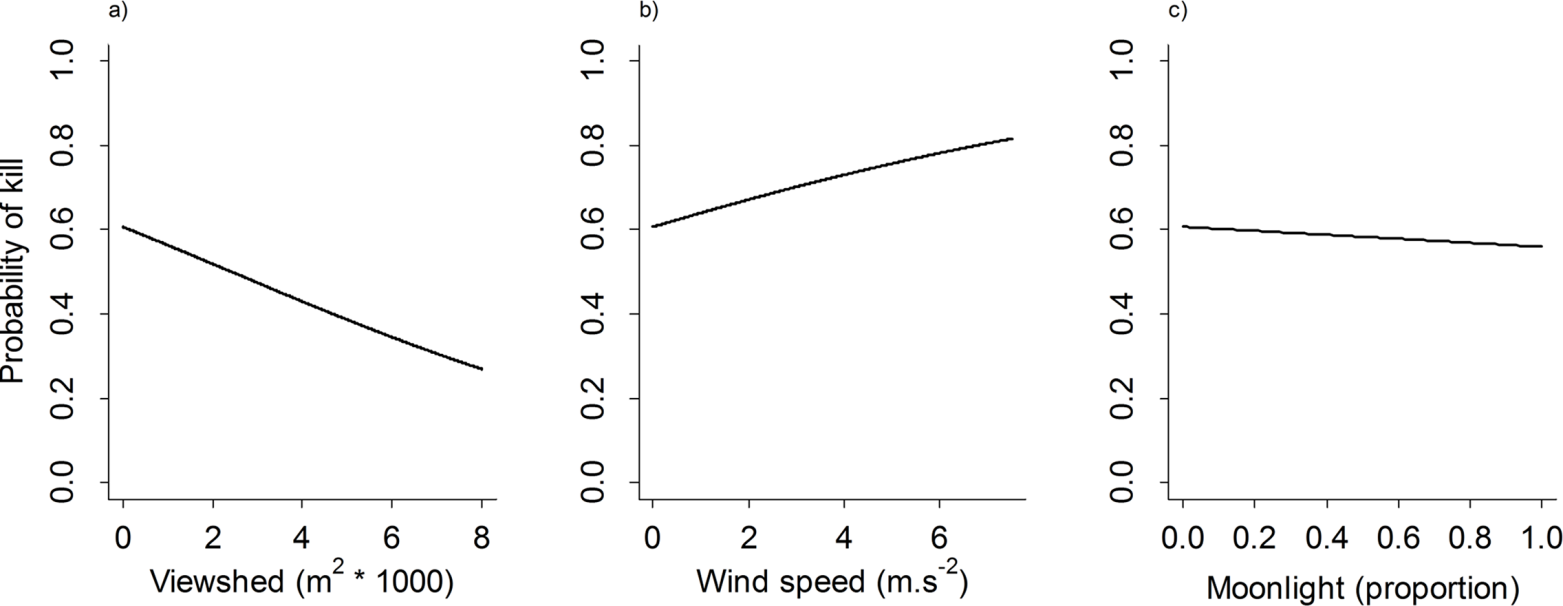
Probability of a site being a kill site for lions (lion sexes combined) in relation to a) viewshed area, b) average wind speed, and c) moonlight. Confidence intervals are too small to be shown due to the large number of iterations (10 000) performed during the analysis.

Lion kill sites were characterized by denser vegetation (smaller viewsheds and shorter distances to cover), and kills occurred during higher wind speeds and darker nights (less moonlight) than resting sites (Fig 3). Of these three variables, viewshed was the most important, featuring prominently in the top models (Table 2) and showing the greatest contrast when compared to resting sites (Fig 3). This was followed by the average wind speed and, less so, by moonlight. Predicted responses generated from the model coefficients supported these findings, with the probability of a site being a kill site decreasing substantially with increasing viewshed (kill site probability was roughly double at the densest sites compared to the most open sites), increasing, although more slowly, with increasing wind speeds and decreasing only slightly with increased moonlight (Table 4, Fig 4). Kill and resting site characteristics for male and female lions were similar (Fig 3), and interactions between lion sex and environmental variables did not feature prominently in the models (Table 2).

**Table 4:** Estimated model averaged coefficients, with standard errors, calculated for each predictor variable present in the models that constituted a cumulative Akaike weight of 0.95, following 10 000 iterations of the random subsampling procedure.

Parameter	Estimate	Standard error
Intercept	0.434	0.002
Viewshed (0–50 m)	-0.179	0.001
Wind speed	0.140	0.001
Moonlight	-0.198	0.002
Lion sex	0.107	0.002
Lion sex: viewshed	-0.014	0.001
Moonlight: lion sex	-0.106	0.004

Although viewsheds around kill sites were smaller than resting sites, random sites within the lion ranges had even smaller viewsheds. Median viewshed differed between all three categories (Kruskal-Wallis, *X*^2^ = 113.7, p < 0.001), with viewsheds of 1498 m^2^ at kill sites, 3813 m^2^ at resting sites and 731 m^2^ at random sites. Pairwise comparisons showed that all three categories differed from each other (p < 0.001 for all comparisons).

### Kill site characteristics across prey species and prey sexes

**Fig 5:**
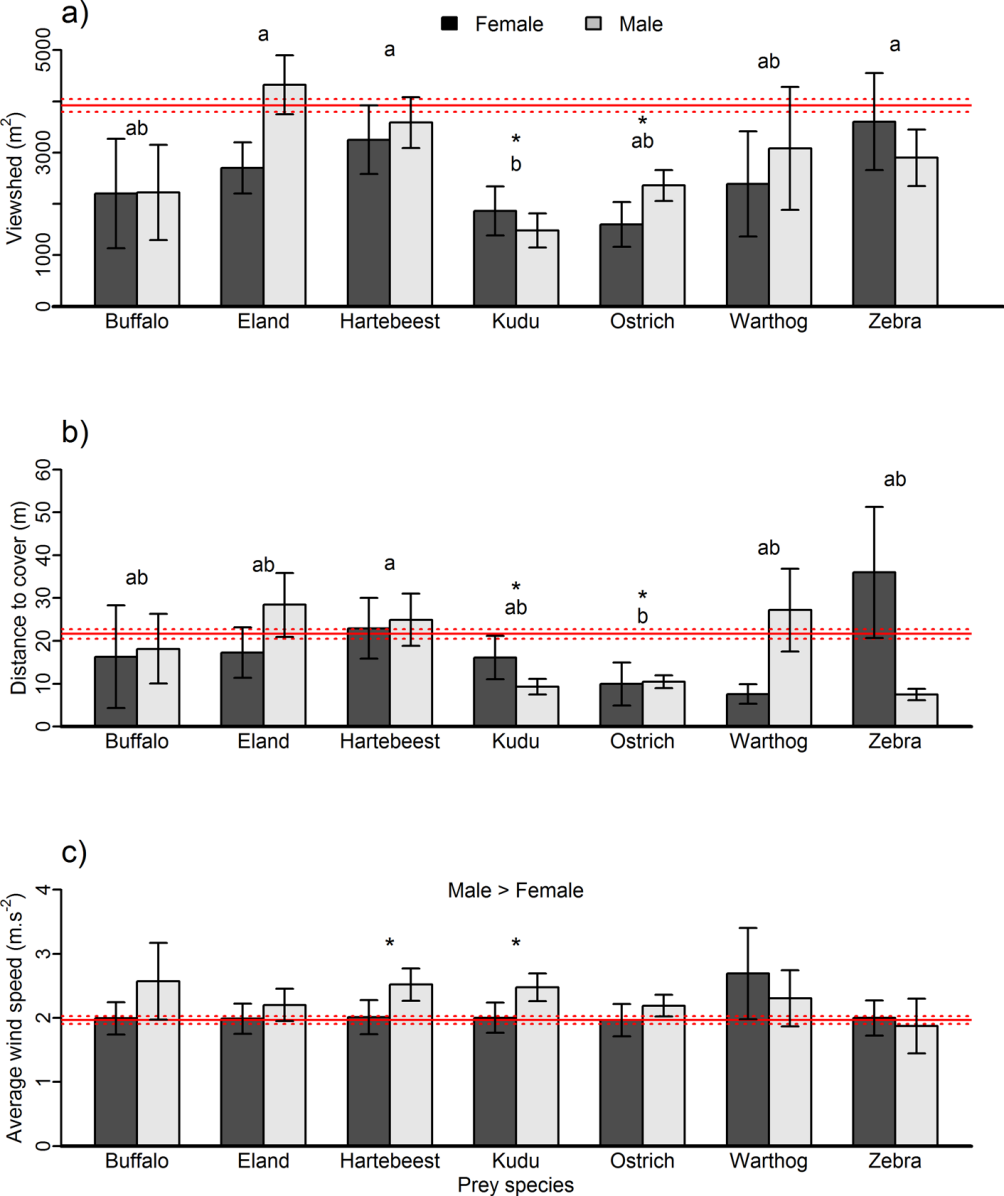
Kill site characteristics for each prey species and prey sex (juvenile prey animals are combined with females) for a) the viewshed, b) the minimum distance to cover (ambush site) in relation to the prevailing wind direction, and c) the average wind speed before the start of the GPS cluster. The solid red line indicates the mean of the characteristic of the non-kill sites; dashed lines represent standard error. Asterisks denote cases where a particular species differed significantly from the non-kill state, whereas letters denote significant differences between species in a) and b). Male prey kill sites were located at sites with significantly higher average wind speeds than female prey (c).

Across prey species, viewsheds at kill sites compared to resting sites were smaller for kudu (mean resting viewsheds: 3919 m^2^, mean kudu kill site viewsheds: 1625 m^2^) and ostrich (mean ostrich kill site viewsheds: 2192 m^2^), whereas they did not differ for the other prey species (Fig 5A). Vegetation around kudu kill sites was also significantly denser (smaller viewsheds) than eland, hartebeest and zebra kill sites. Viewshed area did not differ between prey sexes for any species, although male eland kill sites tended to be in more open locations than females (p = 0.066) (Fig 5A).

**Fig 6:**
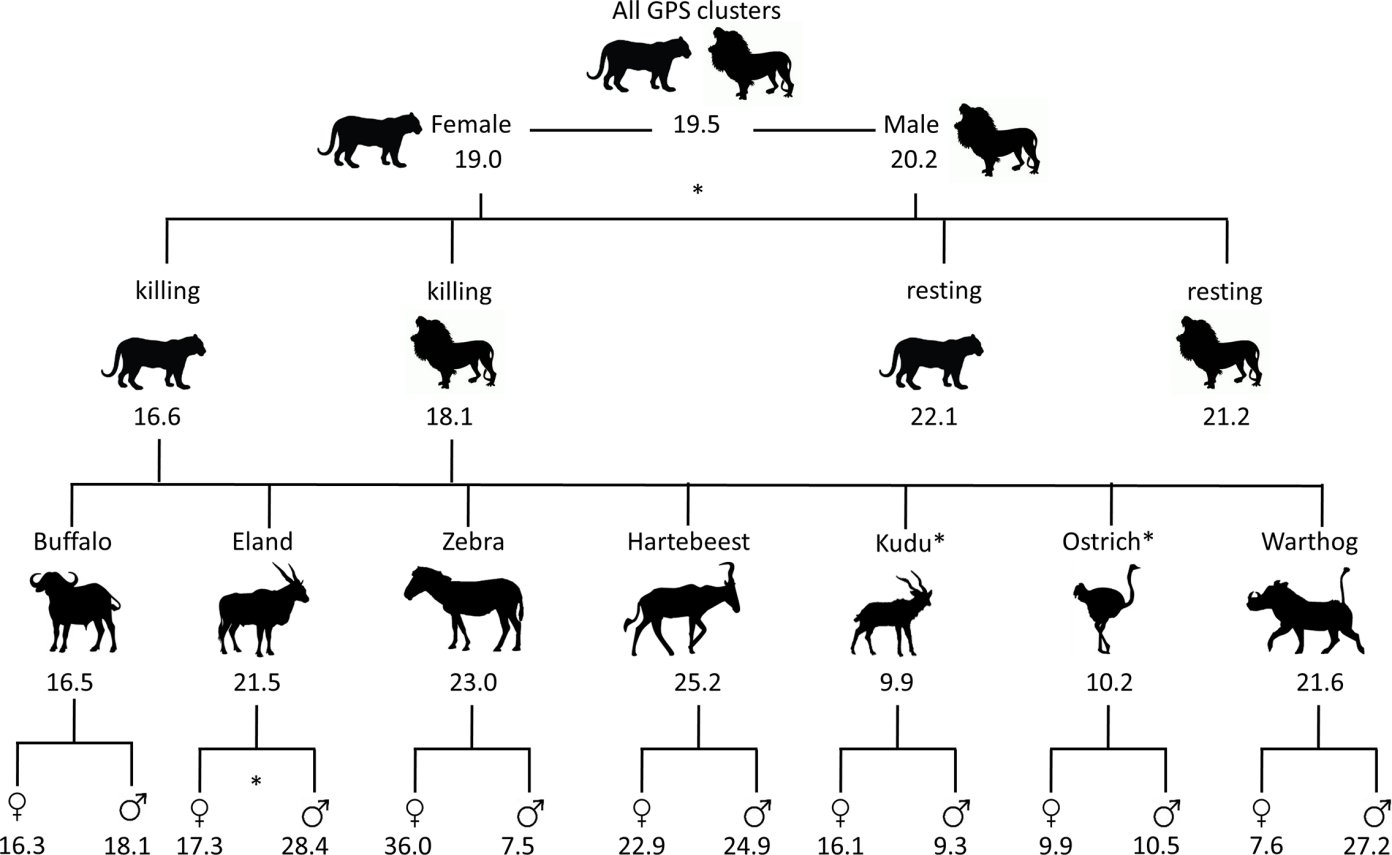
The minimum distance to cover relative to the prevailing wind direction grouped by lion sex, lion behavior (killing or resting), prey species (kill sites), and prey sexes (kill sites for the sex of each prey species). Values for each prey species are taken from the combined measurements across lion sexes and arranged in descending order of prey body size. Asterisks denote significant differences; numbers are mean distance to cover in meters. Although large differences between prey sexes are evident for zebra and warthog, these were not statistically significant due to low sample sizes for those species.

The minimum distance to cover in relation to the prevailing wind direction displayed similar patterns as the viewshed, which was expected due to the collinearity of the variables. We chose to retain this analysis despite the collinearity because it provides a slightly different, but important perspective, especially in the thicket ecosystem of Addo where hard boundaries between dense thicket and open plains occur. Distance to cover was significantly shorter for ostrich (10.2 m from cover) and kudu (9.9 m from cover) kill sites compared to lion resting sites (21.6 m from cover), but in contrast to viewshed, ostrich were killed significantly closer to cover than hartebeest only, and kudu did not differ from any other species in the pairwise comparisons. Male eland were killed significantly further from cover than females (W = 134, p < 0.05, Figs 5B and 6).

The average wind speed during kills for hartebeest and kudu was significantly greater than during lion resting events, but wind speeds at kills did not differ between any of the prey species. Overall, male prey were killed when wind speeds were higher compared to female prey (Fig 5C).

## Discussion

Lions in the Addo ecosystem make the majority of their kills in areas of dense vegetation compared to resting or unsuccessful hunting sites. These conditions provide cover for stalking lions and suggest an ambush style of attack, in keeping with findings for lions elsewhere [19,22]. Although vegetation structure has previously been recognized as an important component of lion hunting success [16,19,36,37], it has been considered less influential relative to other environmental variables [23]. In Addo, however, vegetation density, as measured by viewsheds, was the most important environmental predictor of where lions make kills. However, although lions killed in denser vegetation than where they rested, this was not the densest vegetation in the Addo ecosystem. Many of the random sites were located in even denser vegetation, with limited access to open areas. This suggests an upper limit on vegetation density that provides assistance to hunting lions, with very dense areas not used. The thicket vegetation in Addo can form virtually impenetrable stands, especially in the absence of elephants [42], and lions would struggle to move through the vegetation or locate prey within such areas, rendering them unsuitable for hunting, and possibly providing refuge for some prey species [45].

The finding that viewshed (vegetation structure) exerted the strongest influence over kill site locations, and that it did so equally across lion sexes, could be considered somewhat surprising given the prominence of other factors elsewhere (e.g. [23]) and the strong sexual dimorphism displayed by lions, with sex-based differences in kill locations reported previously [16,23]. However, because our study was not based on direct observations of lion hunting, we cannot definitively conclude that our male lion kill sites were the result of males killing, as opposed to scavenging from females, although our results for male kill (carcass) sites do match results from elsewhere when the same methods were used [16]. To understand the drivers of these differences in lion kill sites, the nature of the Addo ecosystem needs to be considered. Almost all previous lion hunting studies have been conducted in savanna landscapes, and so our understanding of their hunting habits is derived mostly from these ecosystems. In Addo, the succulent thicket vegetation is much denser than typical savanna vegetation, making it feasible for lions to almost always select dense areas as hunting habitats, relying less on other environmental variables such as darker nights. Female lion social groups in Addo also differ from elsewhere. Whereas male groups are comparable to other areas, generally forming coalitions of two or three males, female groups mostly consist of either solitary individuals or females with cubs (C.J. Tambling, pers. obs.), in contrast to pride sociality found in most savanna areas [35]. Our findings for male lion kills are similar to savanna lions (although we might not be recording male lion hunting), but female reliance on vegetation structure is unusual [16]. In savannas, female lion hunting success generally increases with group size [23,36,37], and cooperative hunting is most effective in open areas where predators benefit by encircling prey [23]. In Addo, the cover provided by the dense vegetation negates the need for female lions to form large social groups when hunting. Moreover, hunting success decreases when cubs or sub-adults are present [23], and since the majority of female lion groups larger than singletons in Addo are comprised of mothers with cubs, there is no advantage to hunting in the open, even with increased group size. Female lions in savanna ecosystems also rely on other pride members to safeguard young cubs during group hunts [35]. However, the dense vegetation in Addo likely enables single mothers to hide their cubs more effectively and so be less reliant on forming crèches and therefore groups. Based on our findings, we predict that solitary lionesses will also show such reliance on vegetation structure in savannas, indicating an additional dimension, beyond predator sex, that drives predator behavior. These concepts of varying functional behavior with predator sex and group size should also be assessed for other predators that display sexual dimorphism and varying social structures.

After viewshed, wind speed was the next most important variable for predicting kill sites relative to resting or unsuccessful hunting sites. The dense vegetation lions utilize when hunting in Addo could make increased wind speeds particularly useful because of increased noise during windy conditions (as opposed to more open savanna environments where wind speed has been considered less influential [23] or only important for select prey species [37]). Furthermore, given the coastal location of Addo, strong wind is fairly predictable on a daily basis, being stronger in the afternoon. Such conditions might result in wind speed being more important here than other ecosystems where wind is less prevalent or predictable. High wind speeds were, however, more important for kills of male prey (Fig 5C), possibly because of increased vigilance by female prey with young [40]. How such behavior might affect vulnerability and habitat preference across ungulate sexes will influence the functional roles predators play in ecosystems, with differences in the magnitude of their effect between prey sexes (see [56]).

Although the probability of a site being a kill site and not a resting site increased slightly when the GPS clusters originated during darker periods (less moonlight), moonlight did not feature as an important variable compared to viewsheds or wind speed. Although found to be important elsewhere [23,36], it is likely less influential in Addo because of the dense vegetation. In Kruger National Park, moonlight was considered the most important environmental variable affecting hunting success, but not during impala, *Aepyceros melampus*, hunts [23]. Impala were more often encountered by lions in dense bush [52], where darkness was considered less important because lions could rely on the cover provided by the vegetation [23]. A possible constraint in our methods, however, is that moonlight was taken at the start of the GPS cluster, and we do not know the exact time of the kill and how much moonlight was available then. Because of this, the resolution of our moonlight data is also fairly coarse (moon present or absent), and our results regarding the importance of moonlight should be considered with some caution. However, given our results and the similarity between the different moonlight metrics tested, it is doubtful that moonlight plays a substantive role in lion hunting in Addo.

Not only do the hunting habitats of Addo lions differ from savanna lions, but so do the prey species selected. Whereas buffalo are a preferred prey species in several savanna ecosystems [21,57,58], they constituted less than 5% of kills found during our study, and less than 1% of male kills (Table 5), despite male lions specifically targeting buffalo elsewhere [52]. Although buffalo were initially selected by lions in Addo, they later formed larger, more defensive herds and successfully fended off the majority of lion attacks [15]. The small lion group sizes in Addo possibly prevent successful hunting of large groups of buffalo, which generally requires larger groups of lions [23]. Instead of typical target species such as buffalo and zebra [57], half the kills made in Addo consisted of kudu and ostrich, species which are usually killed in proportion to their abundance (kudu) or actively avoided (ostrich) [57]. Kill sites of these species were located in denser vegetation compared to other prey species (Fig 5A) and lions are possibly selecting them because they are easier to locate (kudu frequent densely vegetated areas) or catch (ostrich are less agile in thick vegetation) in dense vegetation, which is more common in Addo relative to savanna areas. The method we used to detect kill sites (GPS telemetry) does, however, have important biases. Carcass remains likely persist for longer periods in areas of dense vegetation where scavengers might take longer to locate them, and we expect that the remains of small prey species will likely go undetected because of the shorter time required for lions to consume the carcass, diminishing the probability of a GPS cluster forming. Nevertheless, our recorded prey spectrum differs from other studies that employed similar methods [21,22], and large carcasses (such as the species in focus here) are less likely influenced by these limitations [59].

**Table 5:** The proportion of each prey species found at kill sites for male lions, female lions and overall.

Prey species	Male kills	Female kills	Total kills
Buffalo	0.01	0.06	0.04
Eland	0.15	0.12	0.13
Hartebeest	0.15	0.18	0.17
Kudu	0.17	0.25	0.23
Ostrich	0.35	0.23	0.27
Warthog	0.05	0.13	0.10
Zebra	0.12	0.03	0.05

Our results demonstrate that vegetation type (biome) affects the way lions interact with their environment, indicating that effects of vegetation structural heterogeneity on predation patterns are not uniform across ecosystems. Furthermore, we highlight how integral vegetation structure is to predator-prey relationships. In many African ecosystems, vegetation structure is undergoing change. In some places, landscapes are becoming more homogenous through clearing and herbivore impacts. Elephants, for example, are decreasing vegetation density and structural diversity locally [31,32], with direct effects on predation patterns [45]. Alternatively, increased levels of CO_2_ are reducing the stability of several biomes (notably savanna and grassland) and favoring the establishment of forests and denser vegetation [27,28]. As these and similar processes persist, and ecosystems are transformed, predators will likely change their hunting behavior and prey vulnerability will change, with implications for predator and prey conservation and ecosystem stability. Managers need to carefully consider how changes in vegetation structure, including management actions that drive alterations of structure, might help or hinder sustainable predator-prey interactions.

## Supporting Information

S1 Appendix Kill site results using data when only stomach contents were used as the location of a kill site. (DOCX)

S1 Dataset Lion kill and non-kill data. (CSV)

S2 Dataset Lion kill and non-kill data per prey species and prey sex. (CSV)

S1 Fig The mean (± SE) proportion of lion GPS clusters positively identified as kill sites after forensic investigation on foot in relation to the number of days between the origin of the cluster and its investigation. (DOCX)

S2 Fig Mean minimum distance to cover for lion GPS clusters where kills were not found (i.e. resting sites) for each hour of the day. The red rectangle indicates daytime clusters which were excluded from analysis (08:00–17:00) because the minimum distance to cover was lower for resting lions that were likely seeking shade. Both kill and resting sites from these times were excluded.
(DOCX)

S3 Fig Comparisons between kill and non-kill sites using three different moonlight metrics: illumination at the start of the GPS cluster, average illumination before the cluster and minimum illumination before the start of the cluster. (DOCX)

S4 Fig Correlations between various covariates: viewsheds (VS) at various distance bands (0–50m, 50–100m, 100–300m and 0–300m), the minimum distance to cover in relation to the prevailing wind direction (Cover (downwind)) and the minimum distance to cover regardless of the wind direction (Cover (minimum)). The lower panels represent paired scatter plots and the upper panels the corresponding Spearman rank correlation coefficient and p values. Strong correlation is shown between all variables and was further supported by variance inflation factors (VIF). Therefore, only viewshed measurements in the 0–50m distance band were used in the analyses.
(DOCX)
