# Supplementary material for: Effects of Vegetation Structure on the Location of Lion Kill Sites in African Thicket
Source: PLoS One. 2016 Feb 24;11(2):e0149098. doi: 10.1371/journal.pone.0149098 (PMC4766088; doi:10.1371/journal.pone.0149098)
Supplement: S1 Appendix — (DOCX) [file pone.0149098.s001.docx]

**Appendix S1.** Kill site results using data when only stomach contents were used as the location of a kill site.

**Figure 1:** Lion kill and non-kill sites in relation to a) the size of the viewshed, b) the average wind speed before the start of the GPS cluster, c) the distance to the nearest cover (ambush site) relative to the prevailing wind direction and d) the Proportion of moon visible at the start of the cluster of the moon (1 = full moon, 0 = no moon). Shading represents kill sites whereas white indicate non-kill sites. Only kill site data from stomach content remains were used in these analyses.

**Figure 2:** Probability of a site being a kill site for lions (sexes combined) in relation to a) viewshed, b) average wind speed, and c) moon illumination. Only kill site data from stomach content remains were used in these analyses.
