# Supplementary material for: Effects of Vegetation Structure on the Location of Lion Kill Sites in African Thicket
Source: PLoS One. 2016 Feb 24;11(2):e0149098. doi: 10.1371/journal.pone.0149098 (PMC4766088; doi:10.1371/journal.pone.0149098)
Supplement: S1 Fig — (DOCX) [file pone.0149098.s004.docx]

**Fig S1.** The mean (± SE) proportion of lion GPS clusters positively identified as kill sites after forensic investigation on foot in relation to the number of days between the origin of the cluster and its investigation.
