# Supplementary material for: Effects of Vegetation Structure on the Location of Lion Kill Sites in African Thicket
Source: PLoS One. 2016 Feb 24;11(2):e0149098. doi: 10.1371/journal.pone.0149098 (PMC4766088; doi:10.1371/journal.pone.0149098)
Supplement: S2 Fig — The red rectangle indicates daytime clusters which were excluded from analysis (08:00–17:00) because the minimum distance to cover was lower for resting lions that were likely seeking shade. Both kill and resting sites from these times were excluded. (DOCX) [file pone.0149098.s005.docx]

**Fig S2.** Mean minimum distance to cover for lion GPS clusters where kills were not found (i.e. resting sites) for each hour of the day. The red rectangle indicates daytime clusters which were excluded from analysis (08:00 – 17:00) because the minimum distance to cover was lower for resting lions that were likely seeking shade. Both kill and resting sites from these times were excluded.
