# Supplementary material for: Effects of Vegetation Structure on the Location of Lion Kill Sites in African Thicket
Source: PLoS One. 2016 Feb 24;11(2):e0149098. doi: 10.1371/journal.pone.0149098 (PMC4766088; doi:10.1371/journal.pone.0149098)
Supplement: S3 Fig — (DOCX) [file pone.0149098.s006.docx]

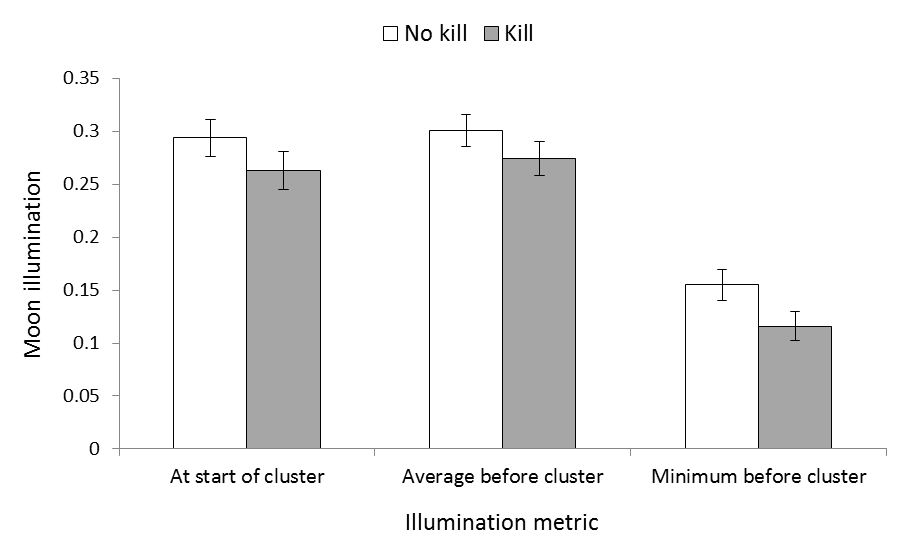


**Figure S3:** Comparisons (mean ± SE) of metrics of moon illumination. Moon illumination at the start of the GPS cluster, average illumination for the hours preceding the cluster and the minimum illumination before the start of the cluster were compared, with similar (and small) differences between kill and non-kill sites recorded for all metrics.
