# Supplementary material for: Effects of Vegetation Structure on the Location of Lion Kill Sites in African Thicket
Source: PLoS One. 2016 Feb 24;11(2):e0149098. doi: 10.1371/journal.pone.0149098 (PMC4766088; doi:10.1371/journal.pone.0149098)
Supplement: S4 Fig — The lower panels represent paired scatter plots and the upper panels the corresponding Spearman rank correlation coefficient and p values. Strong correlation is shown between all variables and was further supported by variance inflation factors (VIF). Therefore, only viewshed measurements in the 0–50m distance band were used in the analyses. (DOCX) [file pone.0149098.s007.docx]

**Fig S4.** Correlations between various covariates: viewsheds (VS) at various distance bands (0 – 50m, 50 – 100m, 100 – 300m and 0 – 300m), the minimum distance to cover in relation to the prevailing wind direction (Cover (downwind)) and the minimum distance to cover regardless of the wind direction (Cover (minimum)). The lower panels represent paired scatter plots and the upper panels the corresponding Spearman rank correlation coefficient and p values. Strong correlation is shown between all variables and was further supported by variance inflation factors (VIF). Therefore, only viewshed measurements in the 0 – 50m distance band were used in the analyses.
